# Antagonizing Interferon-Mediated Immune Response by Porcine Reproductive and Respiratory Syndrome Virus

**DOI:** 10.1155/2014/315470

**Published:** 2014-07-03

**Authors:** Rong Wang, Yan-Jin Zhang

**Affiliations:** ^1^Laboratory Animal Center, School of Medicine, Xi'an Jiaotong University, Xi'an, Shaanxi 710061, China; ^2^Molecular Virology Laboratory, VA-MD Regional College of Veterinary Medicine and Maryland Pathogen Research Institute, University of Maryland, College Park, MD 20742, USA

## Abstract

Interferons (IFNs) are important components in innate immunity involved in the first line of defense to protect host against viral infection. Porcine reproductive and respiratory syndrome virus (PRRSV) leads to severe economic losses for swine industry since being first identified in early 1990s. PRRSV interplays with host IFN production and IFN-activated signaling, which may contribute to the delayed onset and low level of neutralizing antibodies, as well as weak cell-mediated immune response in infected pigs. PRRSV encodes several proteins that act as antagonists for the IFN signaling. In this review, we summarized the various strategies used by PRRSV to antagonize IFN production and thwart IFN-activated antiviral signaling, as well as the variable interference with IFN-mediated immune response by different PRRSV strains. Thorough understanding of the interaction between PRRSV and host innate immune response will facilitate elucidation of PRRSV pathogenesis and development of a better strategy to control PRRS.

## 1. Introduction 

Porcine reproductive and respiratory syndrome (PRRS) is an important infectious disease, causing huge economic losses to the swine industry worldwide [1, 2]. The PRRS clinical signs include respiratory disorders, abortion in pregnant sows, and variable mortality in piglets. PRRS was first identified in the USA in 1987 and subsequently in Europe. The causative agent of the disease is the PRRS virus (PRRSV), a positive-sense single-stranded RNA virus, belonging to the Arteriviridae family in the order Nidovirales [3]. According to the genetic differences, PRRSV is grouped into two genotypes: European (Type 1) and North American (Type 2), represented by Lelystad virus (LV) and VR-2332 strains, respectively.

The genome of PRRSV is about 15 kb in length with 10 open reading frames (ORFs) [3]. ORF1a and ORF1b comprise 80% of the viral genome and encode viral enzymes involved in virus replication. In addition, polypeptides from the two ORFs are processed into 14 nonstructural proteins (nsps), including nsp1*α*, nsp1*β*, nsp2, nsp2TF, and nsp3~12 [3, 4]. ORF2a, ORF2b, ORF3 through ORF7, and ORF5a code for eight structural proteins: GP2, envelop protein (E), GP3~5, membrane protein (M), nucleocapsid protein (N), and ORF5a protein [3, 4].

Swine are the only known host of PRRSV. PRRSV-infected pigs develop a delayed onset of neutralizing antibodies and a weak cell-mediated immune response [5, 6]. The main target cells for PRRSV infection* in vivo* are porcine pulmonary alveolar macrophages (PAMs), which play a crucial role in host immune response [7]. In order to successfully invade host, PRRSV has evolved various strategies to interfere with host innate immunity. Some of the PRRSV proteins take part in the modulation of IFN-mediated immune response.

Host innate immune responses play a key role against early viral infection. Interferons are major components of inmate immunity and have diverse biological functions including antiviral activity, antiproliferative activity, stimulation of T cell cytotoxic activity, and modulation of immune response [8]. There are three types of interferons. In human, type I interferons include IFN-*α*, IFN-*β*, IFN-*ε*, IFN-*κ*, and IFN-*ω* [9, 10]. In addition, IFN-*δ*, IFN-*τ*, and IFN-*ζ* (or Limitin) have been identified as type I IFNs in swine, ruminant, and mice, respectively [11]. Almost all cell types are capable of producing IFN-*α*/*β*; however, plasmacytoid dendritic cells (pDC) are considered to be the major source of IFN-*α* production during viral infection [12, 13]. Type II IFN contains only IFN-*γ*, whose production is restricted to activated T cells, natural killer cells, and macrophages [14]. Type III IFNs comprise IFN-*λ*1, IFN-*λ*2, and IFN-*λ*3 (also known as interleukin- (IL-) 29, IL-28A, and IL-28B, resp.), which are mainly generated by dendritic cells [11]. Considering the major roles in antiviral response by type I IFNs, we focus on this type of IFNs and discuss the PRRSV-mediated interference with their production and signaling.

This review summarizes the recent advances in the research of PRRSV interference with IFN-mediated innate immunity, the viral proteins involved, and their molecular mechanisms, as well as diverse effects by different strains and in different cell types. A few relevant reviews on PRRSV interplay with innate immunity were published previously [15–17]. Readers are encouraged to read them if interested as these reviews were written in different angles to address the issue with diverse scopes, though there is some overlap in certain topics. This review is arranged into sections of IFN induction, IFN-activated signaling, IFN-stimulated genes, and perspective.

## 2. PRRSV Interference with Host Interferon Induction 

Host pattern recognition receptors (PRRs) for RNA viruses include Toll-like receptors (TLRs) and RIG- (retinoic acid inducible gene-) I-like receptors (RLRs). TLRs that can detect viral RNA are TLR3, TLR7, and TLR8 [18]. The RLR family of PRRs comprises RIG-I and melanoma differentiation-associated gene 5 (MDA-5) [19]. Both RIG-I and MDA-5 signal through adaptor IPS-1 (also known as MAVS, Cardif, and VISA) on the outer membrane of the mitochondria [20]. TLR3 and RLR can recognize double-stranded RNA (dsRNA) of viral genome or replication intermediate of RNA viruses. Activation of TLR and RLR signaling pathways leads to activation of interferon regulatory factor 3 (IRF3), IRF7, and NF-*κ*B. These transcription activation factors translocate into the nucleus and result in induction of type I IFNs and expression of inflammatory cytokines, which not only lead to an antiviral state of the neighboring uninfected cells, but also serve as key regulators to evoke adaptive immune response. At least 39 functional type I IFN genes have been identified in porcine chromosomes 1 and 10 [21]. These IFN genes include 17 IFN-*α* subtypes, 1 IFN-*β*, 11 IFN-*δ*, 7 IFN-*ω*, 1 IFN-*α* *ω*, 1 IFN-*ε*, and 1 IFN-*κ*.

PRRSV is sensitive to type I IFNs and the sensitivity is confirmed* in vitro* and* in vivo*. Pretreatment of PAMs with porcine IFN-*α* resulted in significant reduction of PRRSV yield [22]. Pretreatment of MARC-145 cells and porcine pulmonary alveolar macrophages (PAMs) with porcine IFN-*β* inhibited PRRSV replication [23]. Pigs that were inoculated with recombinant adenovirus for IFN-*α* expression and challenged one day later with PRRSV had lower febrile responses, reduced lung lesion, and delayed viremia and antibody response compared to controls [24]. Therefore, for invading host immune clearance, PRRSV has evolved multiple strategies to antagonize the host IFN induction.

### 2.1. PRRSV Inhibition of IFN Induction in Pigs and Cultured Cells

PRRSV appears to inhibit synthesis of type I IFNs in pigs infected with type 1 strains, while swine transmissible gastroenteritis virus (TGEV) and porcine respiratory coronavirus (PRCV) induced high level of IFN-*α* [22, 25]. IFN-*α* could not be detected in the lungs of pigs in which PRRSV actively replicated. It was estimated that the IFN-inducing capacity of PRRSV is at least 159-fold lower than that of PRCV [22].

PRRSV infection of PAMs leads to no IFN-*α* production and when the cells were superinfected with TGEV, no IFN-*α* was detected either [25]. The PRRSV suppression of IFN induction correlates with the virus replication. Plasmacytoid dendritic cells (pDCs) are thought to be the major source of IFN-*α in vivo*. PRRSV fails to induce porcine pDCs to produce IFN-*α*, while pseudorabies virus (PrV), swine influenza virus (SIV), and TGEV stimulated the pDCs to synthesize IFN-*α* [26, 27]. Moreover, presence of PRRSV markedly reduced the typical IFN-*α* response of pDCs to TGEV or Toll-like receptor 9 agonist. Loving et al. showed that PRRSV replicated in monocyte-derived DCs but not lung DCs and the response of both cell types to PRRSV was only limited to IFN-*β* transcription [28]. Additionally, for MARC-145 cells PRRSV replication also significantly inhibited the dsRNA-induced type I IFN expression [29–31]. These data suggest that PRRSV infection directly interferes with type I IFN induction* in vivo* and* in vitro*.

### 2.2. PRRSV Proteins Involved in the Inhibition of IFN Induction

**Figure 1:**
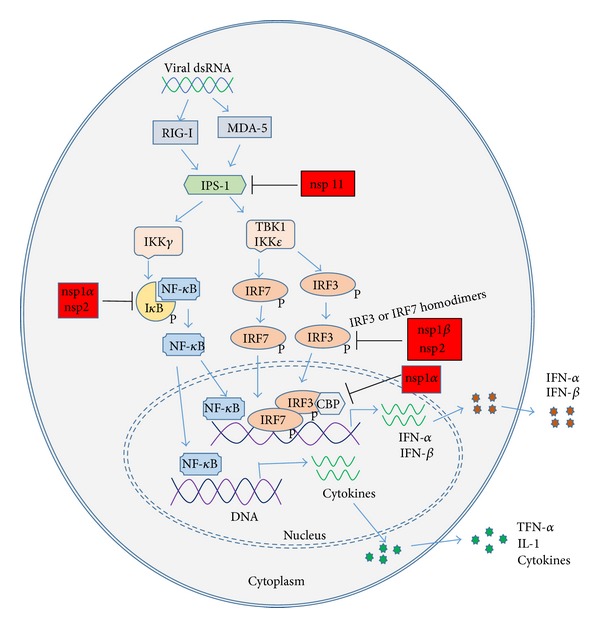
Interference of type I IFN production by PRRSV proteins. Activation of RLR pathway and signaling by viral dsRNA is shown. Viral dsRNA is generated during PRRSV replication. “P” besides IRF3 and IRF7 indicates phosphorylation. Red-colored blocks indicate PRRSV proteins known to inhibit the signaling molecules indicated. PRRSV nsp1*α* inhibits IRF3 association with CBP, enhances CBP degradation, and interferes with I*κ*B degradation. And nsp1*β* inhibits IRF3 phosphorylation and nuclear translocation. Also nsp2 inhibits IRF3 phosphorylation and nuclear translocation, interferes with I*κ*B polyubiquitination, and prevents its degradation. And nsp11 inhibits IRF3 phosphorylation and nuclear translocation via degrading IPS-1 mRNA.

The PRRSV proteins that are found to be antagonists of IFN induction include nsp1, nsp2, nsp11, and N (Figure 1 and Table 1) [30–36]. nsp4, a 3C-like serine protease that is responsible for most of the nonstructural protein processing [37], was found to inhibit IFN-*β* promoter activation in a reporter assay [31], but no further characterization was reported. Further study is needed to elucidate the mechanism.

Also nsp1 has been studied in more detail than others. nsp1 is self-cleaved into nsp1*α* and nsp1*β* subunits, both of which mainly localize in the cell nucleus and dramatically inhibit IFN-*β* expression by affecting the IRF3 signaling pathway [32]. IFN-*β* promoter reporter assay was performed in HEK293T cells in the study. The result showed that nsp1 and its two cleavage products, nsp1*α* and nsp1*β*, inhibited the activation of IFN-*β* promoter (p125-Luc) and an artificial promoter containing three IRF3 binding sites (p55-CIB-Luc) after SV40 stimulation. PRRSV nsp1*α* and nsp1*β* blocked the induction of IFN-*β* at downstream of IRF3 activation but had no effect on the phosphorylation and translocation of IRF3. It suggested that nsp1*α* and nsp1*β* may have a direct effect on the formation of the transcription enhanceosome on the IFN-*β* promoter inside the nucleus. Kim et al. showed that nsp1 inhibited IRF3 association with CREB-binding protein (CBP) in the nucleus but had no effect on IRF3 phosphorylation and nuclear translocation [30]. Immunoprecipitation with anti-IRF3 antibody found that interaction between CBP and IRF3 in PRRSV-infected MARC-145 cells or nsp1-transfected HeLa cells was weaker than in cells treated with polyI:C alone [30]. The expression of nsp1 also enhanced CBP degradation, which can be rescued by MG132 treatment, a proteasome inhibitor. No interaction between nsp1 and CBP was found. The process is independent of the PCP activity of nsp1 [30]. Beura et al. showed that nsp1*β* interfered with IRF3 signaling pathway by inhibiting dsRNA-induced IRF3 phosphorylation and nuclear translocation [31]. The discrepancy is possibly because an nsp1*β* that is 27-residue longer than its authentic form was used in Beura's study. Another possible reason is that different PRRSV strains were used as a couple of other studies showed that PRRSV replication significantly blocked dsRNA-induced IRF3 activation in MARC-145 cells [38, 39]. And nsp1 was also found to downregulate IRF3 protein level and inhibit its phosphorylation [39]. In our laboratory, we observed that PRRSV infection of MARC-145 cells led to reduction of IRF3 protein level (unpublished data). Luo et al. [38] showed PRRSV blocked IFN-*β* production and IRF3 nuclear translocation via significantly inhibiting activation of IPS-1 in RIG-I signaling pathway.

The structure-function studies of nsp1*α* and nsp1*β* identified critical motifs of the proteins in inhibition of IFN induction. The zinc-finger (ZF) domain in the C-terminus of nsp1*α* is critical for this protein to antagonize both IFN-*β* induction and NF-*κ*B activation, especially the 14 amino acids at C-terminal of the nsp1*α* [40]. Shi et al. [41] screened a series of nsp1*α* C-terminal truncated mutants and revealed that the amino acid residue F176 of nsp1*α* is essential for the inhibition of IFN-*β* induction. The residue F176 played a role in both TLR3 signaling and RIG-I signaling pathways [41]. Double mutations K130A/R134A (type 1 PRRSV) or K124A/R128A (type 2 PRRSV) in a highly conserved motif of nsp1*β*, GKYLQRRLQ, dampened the nsp1*β* inhibition of IFN induction [42]. Moreover, recovered recombinant viruses with the nsp1*β* mutations by reverse genetics induced higher level gene expression of type I IFNs than that of wild type viruses.

Also nsp2 inhibits IFN induction by blocking IRF3 phosphorylation and nuclear translocation. The cysteine protease domain (PL2) of nsp2 was necessary for antagonizing activation of IRF3 pathway [33]. The cysteine protease domain (PL2) at the N-terminus of nsp2, which belongs to the ovarian tumor (OTU) protease family, was shown to inhibit type I IFN induction by interfering with the NF-*κ*B signaling pathway [35]. The OTU domain possesses deubiquitinase activity, which interferes with the polyubiquitination of I*κ*B and subsequently prevents its degradation. Recovered recombinant viruses with mutations in the OTU domain of nsp2 by reverse genetics were found to be unable to inhibit NF-*κ*B activation as effectively as the wild type virus.

nsp11 is an endonuclease [43] and IFN antagonist [31]. nsp11 suppressed the activation of IFN-*β* promoter and the expression of IRF3-mediated genes. The endoribonuclease activity of nsp11 was essential for nsp11 to inhibit dsRNA-induced IFN-*β* induction [36]. The amino acid residue H129 of nsp11, a presumed catalytic histidine, was involved in the inhibition of IRF3 phosphorylation. It seems that the inhibition of IRF3 activation is due to the nsp11 endoribonuclease activity, which can cleave mRNA of IPS-1 [15], the adaptor molecule for RIG-I and MDA-5.

The IFN antagonizing activity is not restricted to nonstructural proteins of PRRSV. Structural proteins, such as the N protein, were found to downregulate IFN-*β* mRNA level in polyI:C-treated immortalized PAM cells [34]. N protein interferes with dsRNA-induced phosphorylation and nuclear translocation of IRF3. The multiple components of PRRSV are involved in the interference with IFN induction. The nsps are early proteins and N is a late one, which may play roles at different stages of viral replication.

### 2.3. Variable Effects of Different PRRSV Strains on IFN Induction

The effect of PRRSV replication on IFN induction appears to be variable among different strains and different cell types. PRRSV field isolates have variable suppressive effect on IFN-*α* induction in PAM cultures and the suppression was found at posttranscriptional stage [44]. This is not unexpected as PRRSV strains are divergent in genomic sequences. PRRSV infection of monocyte-derived dendritic cells (Mo-DC) induced the transcription of IFN-*α*/-*β* but no detectable IFN-*α* in culture supernatant, suggesting a blockage at posttranscriptional stage [45]. PRRSV activated the transcription of IFN-*α* in a PI3K-dependent manner in Mo-DC cells. PRRSV infection of MARC-145 cells inhibits IFN gene expression [29] by interfering with the IPS-1 activation in the RIG-I signaling pathway [38]. A variety of type 1 and type 2 PRRSV strains were found to stimulate IFN-*α* secretion by pDC via TLR7 pathway and the effect did not require live virus [46]. The suppressive effect on pDC may be strain dependent.

A novel isolate, A2MC2, induced IFNs in both MARC-145 and PAM cells and virus replication was needed for IFN induction [47]. IFN-*α*2 and elevation of ISGs were detected in A2MC2-infected cells. Sequencing analysis indicated that A2MC2 was closely related to VR-2332 and Ingelvac PRRS MLV with an identity of 99.8% at the nucleotide level [47]. There were a total of 28 nucleotide (nt) variations when compared to VR-2332, resulting in 14 amino acid changes scattered from nt 4681 to the end of the genome. Compared to both VR-2332 and MLV, A2MC2 has 15 unique nucleotides. Yet the mechanism of this strain inducing IFNs is not known and it is intriguing to note that the first 4.6 kb of its genome is identical to VR-2332. Its nsp1*α* and nsp1*β* proteins should be able to inhibit IFN induction. We hypothesize that the unique nucleotides in A2MC2 genome resulting in special RNA structures or unique dsRNA formation during early viral replication could evoke IFN production. It is worth to note that the induction of IFNs is dose dependent. The virus is able to replicate when the inoculum is at less than 0.1 MOI, which induces limited IFNs that cannot suppress the virus replication [47]. A2MC2 infection of pigs resulted in earlier onset and higher level neutralizing antibody against homologous and heterologous strain than MLV vaccine strain that is highly homologous in sequence [48].

### 2.4. PRRSV Interferes with IFN-Activated JAK/STAT Signaling

Type I IFNs are critical to innate immunity against viral infections and play an important role in the stimulation of adaptive immune response [49, 50]. The activation of IFN signaling leads to the induction of antiviral responses. The signaling of type I IFNs is initiated after they bind to their receptors on the cell surface [51–53]. This receptor binding activates Janus kinases (JAK), which phosphorylates both the signal transducers and activators of transcription 1 (STAT1) and STAT2. The phosphorylated STAT1 and STAT2 form heterodimers, followed by interaction with interferon regulatory factor 9 (IRF9) and subsequently formation of heterotrimers, also known as interferon-stimulated gene factor 3 (ISGF3). Translocation of ISGF3 into the nucleus followed by binding to consensus DNA sequences leads to the expression of IFN-stimulated genes (ISGs), which then take part in antiviral responses.

### 2.5. PRRSV Replication Interferes with JAK/STAT Signaling

PRRSV inhibits the IFN-activated JAK/STAT signal transduction and ISG expression in both MARC-145 and PAM cells [54–56]. PRRSV replication in MARC-145 cells suppresses JAK/STAT signaling stimulated by addition of IFN-*α* to the culture [54]. Transcripts of ISG15, ISG56, and protein STAT2 were significantly reduced compared to mock-infected cells. The phosphorylation of both STAT1 and STAT2 was unaffected. Immunoprecipitation with STAT1 or STAT2 antibody for MARC-145 cell lysates was performed and the result showed no significant difference for STAT1/STAT2 heterodimer formation between PRRSV-infected and mock-infected cells. Further study showed that the nuclear translocation of STAT1/STAT2 heterodimer was blocked. PRRSV infection of PAM cells also blocks JAK/STAT signaling shown by reduction of ISG expression after stimulation with external IFN-*α*, while a vaccine strain Ingelvac PRRS MLV had little effect, possible due to its less efficient replication in the primary cells [54].

### 2.6. PRRSV Proteins Involved in the Interference of JAK/STAT Signaling

**Figure 2:**
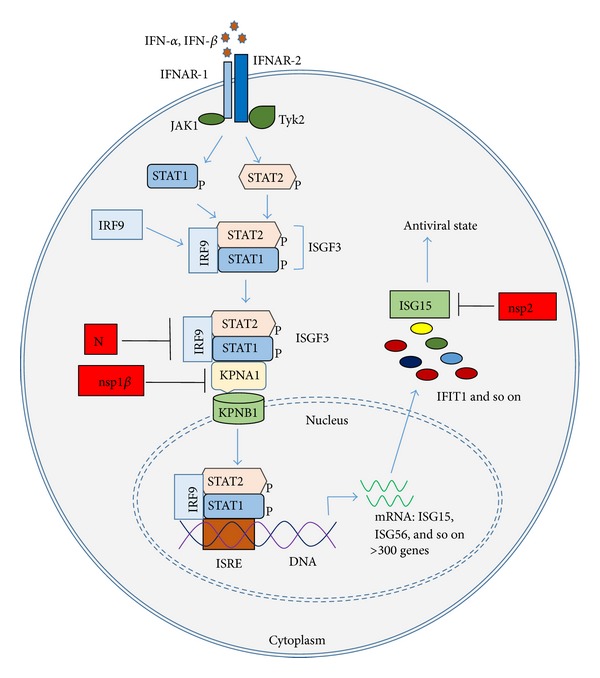
Interference with type I IFN-activated JAK/STAT signaling pathway and antiviral ISGs. IFN-*α*/-*β* binds to their receptors IFNAR-1 and IFNAR-2 on cell membrane, which activates JAK/STAT pathway. “P” besides STAT1 and STAT2 indicates phosphorylation. PRRSV nsp1*β* inhibits ISGF3 nuclear translocation via inducing degradation of KPNA1, which is essential for mediating the nuclear import of ISGF3. N protein also inhibits ISGF3 nuclear translocation. And nsp2 reduces ISG15 production and conjugation via its deubiquitination activity.

PRRSV proteins that interfere with IFN-activated signaling include nsp1*β*, nsp7, nsp12, GP3, and N (Figure 2 and Table 1) [54–56]. PRRSV nsp1*β* was found to block the nuclear translocation of STAT1 and significantly inhibit the expression of ISGs [54]. By observing STAT1-GFP distribution under fluorescence microscopy, Chen et al. [32] noticed that STAT1-GFP accumulated in cytoplasm in HEK293T cells with nsp1*β* expression. Further studies on nsp1*β* revealed that it induced the degradation of karyopherin-alpha1 (KPNA1, also called importin-alpha5), which is known to mediate the nuclear import of ISGF3 [56]. The N-terminal domain of nsp1*β* was involved in the ubiquitin-proteasomal degradation of KPNA1. Residue 19 of nsp1*β* was found to be essential in inducing KPNA1 degradation and inhibiting IFN-mediated signaling as the residue change from valine to isoleucine diminished the suppressive effect. Notably, PRRSV infection of MARC-145 cells by VR-2332 and VR-2385 also reduces KPNA1 expression, whereas infection by Ingelvac PRRS MLV does not. MLV nsp1*β* has no effect on KPNA1 expression or IFN signaling but gains the suppressive function when residue 19 is changed to valine [56].

Other PRRSV proteins including nsp7, nsp12, GP3, and N were also found to be able to inhibit IFN-activated signaling [55]. N protein inhibits IFN-activated STAT1 nuclear translocation, albeit less effective than nsp1*β* [55].

### 2.7. Variable Effect of Different PRRSV Strains on the IFN-Activated Signaling

Variable effect on IFN signaling among PRRSV strains was found [55]. Among six PRRSV strains (VR-2385, Ingelvac PRRS MLV, VR-2332, NVSL97-7895, MN184, and Lelystad) tested, all but MN184 inhibited IFN signaling in MARC-145 cells, and all but MLV and NVSL97-7895 blocked the IFN activation in PAMs. The result also demonstrated that the interference with IFN signaling by PRRSV was variable in different infected-cell types. nsp1*β* from the six strains was cloned and all but MLV nsp1*β* inhibited IFN signaling when overexpressed [55]. The IFN-inducing A2MC2 strain has no effect on JAK/STAT signaling activated by IFN-*α* [47].

## 3. PRRSV Interference with the Function of Antiviral ISGs

Type I IFNs (e.g., IFN-*α* and IFN-*β*) drive the expression of more than 300 genes that encode proteins with antiviral, antiproliferative, proapoptotic, and proinflammatory functions [57]. Among the antiviral ISGs, the best studied ones are 2′,5′-oligoadenylate synthetases (OASs), ribonuclease L RNaseL, the dsRNA-activated protein kinase (PKR), p56 (ISG56, interferon-induced protein with tetratricopeptide repeats 1 (IFIT1)), Mx1 (Myxovirus (influenza virus) resistance 1), and ISG15.

PRRSV nsp2 was shown to inhibit the antiviral function of IFN-stimulated gene 15 (ISG15) by the deubiquitinase activity of the OTU domain of nsp2 (Figure 2 and Table 1) [58]. ISG15 is an ubiquitin-like antiviral protein [59, 60]. ISG15 conjugation (ISGylation) to substrate proteins follows a process similar to that of ubiquitin conjugation. ISGylation of many important immune-related molecules leads to the activation of host innate immune response. As mentioned above, the cysteine protease domain (PL2) at the N-terminus of nsp2 belongs to OTU-containing superfamily of proteases (DUBs), which possesses deubiquitinating activity [58]. Sun et al. revealed that nsp2 was an antagonist for the antiviral activity of ISG15 by reducing ISG15 production and conjugation. The N-terminal PL2 domain of nsp2 was crucial for the antagonizing function.

PKR mediates translational control by phosphorylating the protein translation initiation factor eIF2*α*, resulting in inhibition of protein synthesis and viral replication [61]. Addition of 2-aminopurine (2-AP), an inhibitor of PKR, restored PRRSV replication in IFN-*γ*-treated cells [62]. Addition of 2-AP to recombinant swine IFN-*β*-primed MARC-145 cells restored PRRSV replication but did not rescue the virus in IFN-*β*-primed PAM cells [63]. These results showed the important role of PKR in the IFN-activated antiviral signaling. We found that PRRSV is able to inhibit the polyI:C-induced activation of PKR, as well as its downstream effector eIF2*α* (unpublished observation).

It is not known if PRRSV interferes with other ISGs. Considering the important roles of the ISGs in deterring invading pathogens, one can imagine that PRRSV must have evolved strategies to evade them during its replication. Further study on the interplay of PRRSV and ISGs will provide insights into such strategies.

## 4. Perspective

PRRSV infection in pigs leads to delayed production and low titer of neutralizing antibodies [5], as well as weak cell-mediated immune response [6]. Partly the reason is possibly because of PRRSV interference with IFN-mediated innate immunity. PRRSV infection appears to inhibit synthesis of type I IFNs* in vivo* and* in vitro* with the exception of some atypical strains that induce IFN production, such as A2MC2. The mechanism for the interference is at multiple steps from inhibition of IRF3 activation, CBP interaction with IRF3, and posttranscriptional suppression. PRRSV also block IFN-activated signaling, which results in suppression of the expression of antiviral ISGs. The mechanism is PRRSV-mediated blocking of ISGF3 nuclear translocation.

The PRRSV interference with the innate immunity is at multiple levels, from IFN induction and IFN-activated signaling to activity of ISGs. Given the divergence of PRRSV strains in sequences, variation of these activities in innate immunity is not a surprise, whereas the multifold interplay between the virus and host may determine the consequences. In addition, type I IFNs are proinflammatory cytokines. The protective effect of IFNs* in vivo* may be context dependent. The IFNs in proper amount at right site and time should be protective. Otherwise, its elevation might be a consequence of inflammation during PRRSV infection. A typical example is that high-pathogenic PRRSV induces high level IFN-*α* but causes high mortality in pigs [64]. Further study is needed to elucidate the contribution of PRRSV effect on innate immunity to its pathogenesis and the modulation of adaptive immune responses.

## Figures and Tables

**Table 1:** PRRSV viral proteins interfering with IFN induction and signaling.

Protein	Target	Mechanisms	References
nsp1 (nsp1*α* and nsp1*β*)	IFN induction	Interfering with IRF3 phosphorylation and nuclear translocation	[31]
		Inhibiting IRF3 association with CBP and enhancing CBP degradation	[30]
		Inhibiting IFN-*β* induction at downstream of IRF3 activation	[32]
		Downregulating IRF3 and inhibiting its phosphorylation	[39]

nsp1*β*	IFN-activated signaling	Blocking STAT1/STAT2 nuclear translocation via inducing KPNA1 degradation	[54–56]

nsp2	IFN induction	Blocking IRF3 phosphorylation and nuclear translocation	[33]
		Interfering with I*κ*B polyubiquitination and preventing its degradation	[35]
	ISG15	Reducing ISG15 production and conjugation by nsp2 deubiquitinase activity	[58]

nsp11	IFN induction	Inhibiting IRF3 phosphorylation and nuclear translocation	[36]
		Degrading IPS-1 mRNA	[15]

N	IFN induction	Blocking IRF3 phosphorylation and nuclear translocation	[34]
	IFN signaling	Blocking STAT1/STAT2 nuclear translocation	[55]
